# 2B4 costimulatory domain enhancing cytotoxic ability of anti-CD5 chimeric antigen receptor engineered natural killer cells against T cell malignancies

**DOI:** 10.1186/s13045-019-0732-7

**Published:** 2019-05-16

**Authors:** Yingxi Xu, Qian Liu, Mengjun Zhong, Zhenzhen Wang, Zhaoqi Chen, Yu Zhang, Haiyan Xing, Zheng Tian, Kejing Tang, Xiaolong Liao, Qing Rao, Min Wang, Jianxiang Wang

**Affiliations:** 1 State Key Laboratory of Experimental Hematology, Institute of Hematology and Blood Diseases Hospital, Chinese Academy of Medical Sciences & Peking Union Medical College, 288 Nanjing Road, Tianjin, 300020 China; 2 National Clinical Research Center for Blood Diseases, Institute of Hematology and Blood Diseases Hospital, Chinese Academy of Medical Sciences & Peking Union Medical College, 288 Nanjing Road, Tianjin, 300020 China

**Keywords:** CD5, CAR, NK, Immunotherapy, T cell malignancies

## Abstract

**Background:**

Chimeric antigen receptor engineered T cells (CAR-T) have demonstrated extraordinary efficacy in B cell malignancy therapy and have been approved by the US Food and Drug Administration for diffuse large B cell lymphoma and acute B lymphocytic leukemia treatment. However, treatment of T cell malignancies using CAR-T cells remains limited due to the shared antigens between malignant T cells and normal T cells. CD5 is considered one of the important characteristic markers of malignant T cells and is expressed on almost all normal T cells but not on NK-92 cells. Recently, NK-92 cells have been utilized as CAR-modified immune cells. However, in preclinical models, CAR-T cells seem to be superior to CAR-NK-92 cells. Therefore, we speculate that in addition to the short lifespan of NK-92 cells in mice, the costimulatory domain used in CAR constructs might not be suitable for CAR-NK-92 cell engineering.

**Methods:**

Two second-generation anti-CD5 CAR plasmids with different costimulatory domains were constructed, one using the T-cell-associated activating receptor-4-1BB (BB.z) and the other using a NK-cell-associated activating receptor-2B4 (2B4.z). Subsequently, BB.z-NK and 2B4.z-NK were generated. Specific cytotoxicity against CD5^+^ malignant cell lines, primary CD5^+^ malignant cells, and normal T cells was evaluated in vitro. Moreover, a CD5^+^ T cell acute lymphoblastic leukemia (T-ALL) mouse model was established and used to assess the efficacy of CD5-CAR NK immunotherapy in vivo.

**Results:**

Both BB.z-NK and 2B4.z-NK exhibited specific cytotoxicity against CD5^+^ malignant cells in vitro and prolonged the survival of T-ALL xenograft mice. Encouragingly, 2B4.z-NK cells displayed greater anti-CD5^+^ malignancy capacity than that of BB.z-NK, accompanied by a greater direct lytic side effect versus BB.z-NK.

**Conclusions:**

Anti-CD5 CAR-NK cells, particularly those constructed with the intracellular domain of NK-cell-associated activating receptor 2B4, may be a promising strategy for T cell malignancy treatment.

**Electronic supplementary material:**

The online version of this article (10.1186/s13045-019-0732-7) contains supplementary material, which is available to authorized users.

## Background

The prognoses of patients with T cell malignancies remain poor [1–4]. There is no better treatment strategy than chemotherapy, which may not benefit refractory/relapsed patients and can lead to serious toxicity. It is thus imperative that novel effective targeted therapeutic strategies are developed. In recent years, chimeric antigen receptor (CAR)-modified immune cells have shown outstanding efficacy for the treatment of B cell malignancies [5, 6]. This indicated that using similar concepts to develop CAR-modified immune cells may help fight against T cell malignancies.

Conventional CAR immunotherapy utilizes modified T cells derived from patients to directly target and eliminate malignancies [6]. However, malignant T cells may have the same phenotypic and functional characteristics as normal T cells. This leads to difficulties in distinguishing therapeutic CAR engineered T (CAR-T) cells from malignant T cells, causing the mutual killing of CAR-T cells and limiting the function of CAR-T cells against T cell malignancies [7]. Mamonkin et al. constructed CAR-T cells targeting CD5^+^ T malignant cells and found that the delayed initial expansion of anti-CD5 CAR-T cells was mainly due to fratricide mediated by perforin secretion. [8]. Pinz et al. used anti-CD4 CAR-T cells to eliminate CD4^+^ T cell lymphomas (TCLs) or T cell acute lymphoblastic leukemia (T-ALL), demonstrating that almost all CD4^+^ CAR-T cells were also depleted. A recent study demonstrated that CD4^+^ CAR-T cells might have a “helper effect”, which could enhance the persistence and cytotoxicity of CD8^+^ CAR-T cells [9]. Thus, the self-killing of CD4^+^ CAR-T cells would decrease the cytotoxic ability of CAR-T cells. Moreover, circulating malignant T cells are often found in the peripheral blood of patients with T-ALL [10, 11] and some TCLs [12], which may lead to contamination of malignant T cells and then generate “CAR-malignant T cells” during the process of CAR-T cells preparation [7]. Ruella et al. reported a relapse in a patient after 9 months of anti-CD19 CAR-T cell treatment. The relapsed leukemia cells were CD19 negative, but anti-CD19 CAR was aberrantly expressed. They found that the CAR gene was accidentally transduced into a single B malignant cell during the process of CAR-T cell preparation, and its product concealed the CD19 epitope on the surface of leukemic cells, masking their recognition by CAR-T cells [13]. Similarly, the occurrence of “CAR-malignant T cells” may lead to disease relapse and adversely affect the prognosis of patient with T-ALL and TCL. Therefore, when targeting T-malignant cells, it is necessary to try other types of effector cells to circumvent the shortcomings of CAR-T cells.

Recently, another immune cell, the natural killer (NK) cell, has been used to engineer with CAR [14, 15]. The use of NK cells for CAR-NK cell manufacturing is a promising strategy to avoid mutual killing of CAR-T cells in the abovementioned situation. NK cells are an important part of the innate immune system and have natural cytotoxic ability against malignant cells. NK cells serve as allogeneic effectors, mediating their activity independent of major histocompatibility complexes. Therefore, NK cells do not need to be collected from a certain patient or a specific human leukocyte antigen matched donor to naturally induce graft-versus-host disease [16]. Unfortunately, NK cells from peripheral blood are difficult to transduce with CAR [17]. In our preliminary experiments, we tried different methods to improve the transfection efficiency of NK cells from peripheral blood, including increasing the lentivirus titer, but these failed and led to proliferation inhibition and apoptosis induction in NK cells. As a NK cell line, NK-92 cells have been used as effector cells in immunotherapy, but are derived from a patient with NK cell lymphoma and need to be irradiated before being administered into patients to prevent potential carcinogenicity. Recently, several studies have revealed that NK-92 cells (not transduced with CAR) are safe and effective for the treatment of relapsed/refractory hematological malignancies [18, 19].

CD5 is a type-I transmembrane glycosylated protein [20] that has a role in negative regulation of T cell receptor signaling [21, 22] and promotes the survival of normal and malignant lymphocytes [23, 24]. CD5 is not expressed on the surface of hematopoietic stem cells but is highly expressed by malignant T cells [25, 26]. Therefore, CD5 is currently considered one of the characteristic antigens of malignant T cells [8]. In addition, CD5 is also expressed in some B cell malignancies [27, 28]. Clinical trials using anti-CD5 monoclonal antibody have revealed a moderate therapeutic effect in patients with cutaneous T cell lymphomas (CTCLs) or chronic lymphocyte leukemia (CLL) [29, 30]. Chen et al. designed a third-generation anti-CD5 CAR construct with the T-cell-associated costimulator 4-1BB and CD28 to generate anti-CD5 CAR-NK-92 cells, which showed specific cytotoxicity against CD5^+^ malignant cells in vitro and in vivo [15].

However, at least in preclinical models, it appears that CAR-T cells seem to be superior to CAR-NK-92 cells [7]. CARs commonly contain three domains: an extracellular antigen binding domain, a transmembrane module, and an intracellular signaling transduction domain [31]. The transmembrane module primarily anchors the CAR structure on the cell membrane and is usually driven from the transmembrane region of CD8 or CD28. The classical intracellular signal transduction domain contains a CD3ζ cytoplasmic domain and one or more intracellular domains of costimulatory molecules, such as 4-1BB, CD28, OX40, or ICOS [32]. Different costimulatory domains endow CAR-T cells with different characteristics: a CD28 costimulatory domain stimulates more powerful cytotoxic ability of CAR-T cells, whereas the 4-1BB and ICOS costimulatory domain induces longer persistence of CAR-T cells [9]. All of these costimulatory factors play important roles in the activation and function of T cells. Therefore, we hypothesized that NK-cell-associated costimulatory factors could be used to activate NK cells and exert their cytotoxic effects.

We speculated that the NK-cell-associated costimulatory domain used in a CAR construct might be suitable for engineered CAR-NK-92 cells. Recently, Li et al. used transmembrane domains and costimulatory domains typically expressed in NK cells to construct CARs and found that CAR with a NKG2D transmembrane domain and 2B4 costimulatory domain displayed superior anti-ovarian cancer activity [14]. 2B4 is considered a NK-cell-specific costimulatory receptor belonging to the signaling lymphocytic activation molecule (SLAM) family, which transduces activation signals through SLAM-associated protein (SAP). SAP interacts with the intracellular domain of 2B4 and regulates 2B4-dependent NK cell activation [33, 34].

In this study, the anti-CD5 single-chain variable fragment (scFv) domain of CAR was developed from a mouse anti-human CD5 monoclonal antibody (Clone HI211) that was previously established and validated in our institute. Two different anti-CD5-CARs with costimulators 4-1BB and 2B4 (referred to as BB.z-NK and 2B4.z-NK, respectively) were constructed. Their cytotoxic ability was evaluated, demonstrating that 2B4.z-NK cells exhibited rapid proliferation and higher anti-malignant efficacy in both malignant CD5^+^ cell lines and primary CD5^+^ malignant cells in vitro through upregulation of activation markers and cytotoxic granule release. Furthermore, the superior cytotoxic ability of 2B4.z-NK against T-ALL was confirmed in mouse xenograft models. In addition, both BB.z-NK and 2B4.z-NK have side effects on CD5^+^ normal T cells. To our knowledge, there has been no previous research describing such a strategy of using the 2B4 costimulatory domain to generate anti-CD5 CAR-NK cells for CD5^+^ malignancy treatment.

## Methods

### Patients and samples

Peripheral blood from healthy donors was acquired from the Tianjin Blood Center. Bone marrow samples were obtained from patients enrolled in the Institute of Hematology and Blood Diseases Hospital, Chinese Academy of Medical Sciences, and patient samples were approved by the ethical advisory board of the Institute of Hematology and Blood Diseases Hospital. All subjects signed an informed consent in accordance with the Declaration of Helsinki.

### Plasmid construction and lentivirus production

The murine anti-human CD5 scFv derived from mouse hybridoma cells (clone HI211, which was established in our institute) was cloned into a previously constructed pCDH-CAR plasmid containing the 4-1BB costimulatory domain [35] to form a plasmid referred to as BB.z. Then, the 2B4 intercellular domain was used to replace the 4-1BB costimulatory domain of BB.z to construct a pCDH-CD5 scFv-CD8α hinge-CD8α transmembrane domain-2B4 costimulatory domain-CD3ζ plasmid (referred to as 2B4.z).

Lentiviral vectors were produced in 293T cells as previously described [36].

### Cell culture

Jurkat, MOLT-4, MAVER-1, 293T, and NK-92 cells were purchased from American Type Culture Collection. Jurkat, MOLT-4, and MAVER-1 cells were maintained in RPMI-1640 medium supplemented with 10% fetal bovine serum (FBS). 293T cells were maintained in Dulbecco’s modified Eagle’s medium supplemented with 10% FBS and glutaMAX (GIBCO, USA). NK-92 cells were grown in α-minimum essential medium supplemented with 0.2 mM inositol, 0.1 mM 2-mercaptoethanol, 0.02 mM folic acid, 200 U/ml recombinant human IL-2 (rhIL-2), 12.5% horse serum, and 12.5% FBS. MV4-11 cells were grown in Iscove’s modified Dulbecco’s medium (IMDM) supplemented with 10% FBS. Primary patients’ bone marrow mononuclear cells (BMMNCs) were seeded in IMDM supplemented with 15% FBS, 100 ng/ml rhFLT3-L, 100 ng/ml rhSCF, and 50 ng/ml rhTPO. Primary normal T cells were isolated and cultured as previously described [36].

### Establishment of stable cell lines

Jurkat cells were infected with lentivirus carrying pLV-luciferase-neo plasmid, which was kindly provided by Dr. Rong Xiang (Medical School of Nankai University, Tianjin, China), followed by clonal selection using 600 μg/ml G418 to generate stable polyclonal cells overexpressing firefly luciferase (Jurkat-luc2).

NK-92 cells were infected with lentivirus carrying BB.z CAR plasmid, 2B4.z CAR plasmid, or empty vector, followed by sorting of GFP and F (ab’)_2_-positive cells by flow cytometry to generate polyclonal cells stably expressing BB.z CAR (BB.z-NK), 2B4.z CAR (2B4.z-NK), or VEC-NK cells.

### Cell proliferation assay

We seeded 1.5 × 10^4^ NK cells in 96-well plates per well. After 24 h, 48 h, or 72 h incubation, cell activity was tested by applying Cell Counting Kit-8 (Dojindo, Japan) following the manufacturer’s instructions.

### Apoptosis assay

We then harvested 5 × 10^5^ NK cells and stained with Annexin V-Alexa Fluor® 647 and PI (Biolegend, USA) following the manufacturer’s instructions and then subjected cells to flow cytometry analysis (BD LSRFortessa, USA).

### In vitro function studies of CAR-NK cells

Jurkat, MOLT-4, and MAVER-1 cells were used as CD5^+^ target cells and MV4-11 cells were used as CD5^−^ target cells. Three donors’ normal T cells were used as target cells to evaluate the side effects of CAR-NK cells.

BB.z-NK and 2B4.z-NK cells were used as effector cells and VEC-NK cells as controls.

#### Analysis of direct cytotoxicity

BB.z-NK, 2B4.z-NK, or VEC-NK cells were incubated with target cells at E:T ratios of 4:1, 2:1, 1:1, 1:2, 1:4, or 1:8. After 6 h, the cell mixture was harvested and stained with APC-conjugated anti-human CD5 antibody and PE-Cy7-conjugated anti-human CD56 antibody (Biolegend, USA) for 30 min at 4 °C, and then washed and resuspended in PBS for flow cytometry analysis. The percentage of CD56^−^CD5^+^ cells represented the residual level of target cells.

#### Cytokine releasing assay

BB.z-NK, 2B4.z-NK, or VEC-NK cells were cocultured with target cells at E:T ratios of 1:1 for 12 h. The supernatant of the cocultured system was harvested. Expression levels of IFN-γ and TNF-α were detected using an ELISA kit (R&D, USA) according to the manufacturer’s instructions.

#### Degranulation assay

We cocultured 0.5 × 10^5^ BB.z-NK, 2B4.z-NK, or VEC-NK cells with 1.5 × 10^5^ target cells in 200 μl of NK-92 cultured medium with PE-conjugated anti-CD107a antibody (Biolegend, USA). After 1 h, 100 μg/ml monensin (BD Biosciences) was added to the cocultured system and incubated for 4 h, and then the cells were labeled with PE-Cy7-conjugated anti-human CD56 antibody and analyzed by flow cytometry. All CD56^+^ CD107a^+^ cells were regarded as degranulated NK cells.

#### Detection of NK cell activation markers

BB.z-NK, 2B4.z-NK, or VEC-NK cells were incubated with MAVER-1 cells at E:T ratios of 1:1. After 6 h, cells were harvested and stained with PE-conjugated anti-human CD69 antibody, APC-Cy7-conjugated anti-human HLA-DR antibody, and PE-conjugated anti-human NKG2D antibody (Biolegend, USA) for 30 min at 4 °C, and then washed and resuspended in PBS for flow cytometry analysis. The percentage of CD56^+^CD69^+^, CD56^+^HLA-DR^+^, or CD56^+^NKG2D^+^ cells represented the activated NK cells.

### In vivo NSG murine studies

Eight-week-old NSG female mice were purchased from the Institute of Laboratory Animal Sciences (CAMS&PUMC, China). All animal experiments were approved by the Institutional Animal Care and Use Committee of Peking Union Medical College.

Twenty-four mice were intravenously inoculated with 3 × 10^6^ Jurkat-luc2 cells. Nine days after transplantation, mice were randomly divided into four treatment groups according to the average radiance of bioluminescent imaging: group PBS, group VEC-NK, group BB.z-NK, and group 2B4.z-NK. Mice were intravenously administered PBS or 5 × 10^6^ cells of either VEC-NK, BB.z-NK, or 2B4.z-NK cells at day 10, day 20, and day 26. Bioluminescent images were obtained using Caliper IVIS Lumina II (Caliper Life Sciences, USA), and the average radiance was calculated as described before [37].

### Statistical analyses

Values were expressed as the mean ± S.D. If not specifically mentioned, the statistical significance of data was assessed by an unpaired two-tailed *t*-test. A value of *p* < 0.05 was used as the standard for statistical significance.

## Results

### Construction of CD5 CAR and preparation of CAR-NK cells

**Fig. 1:**
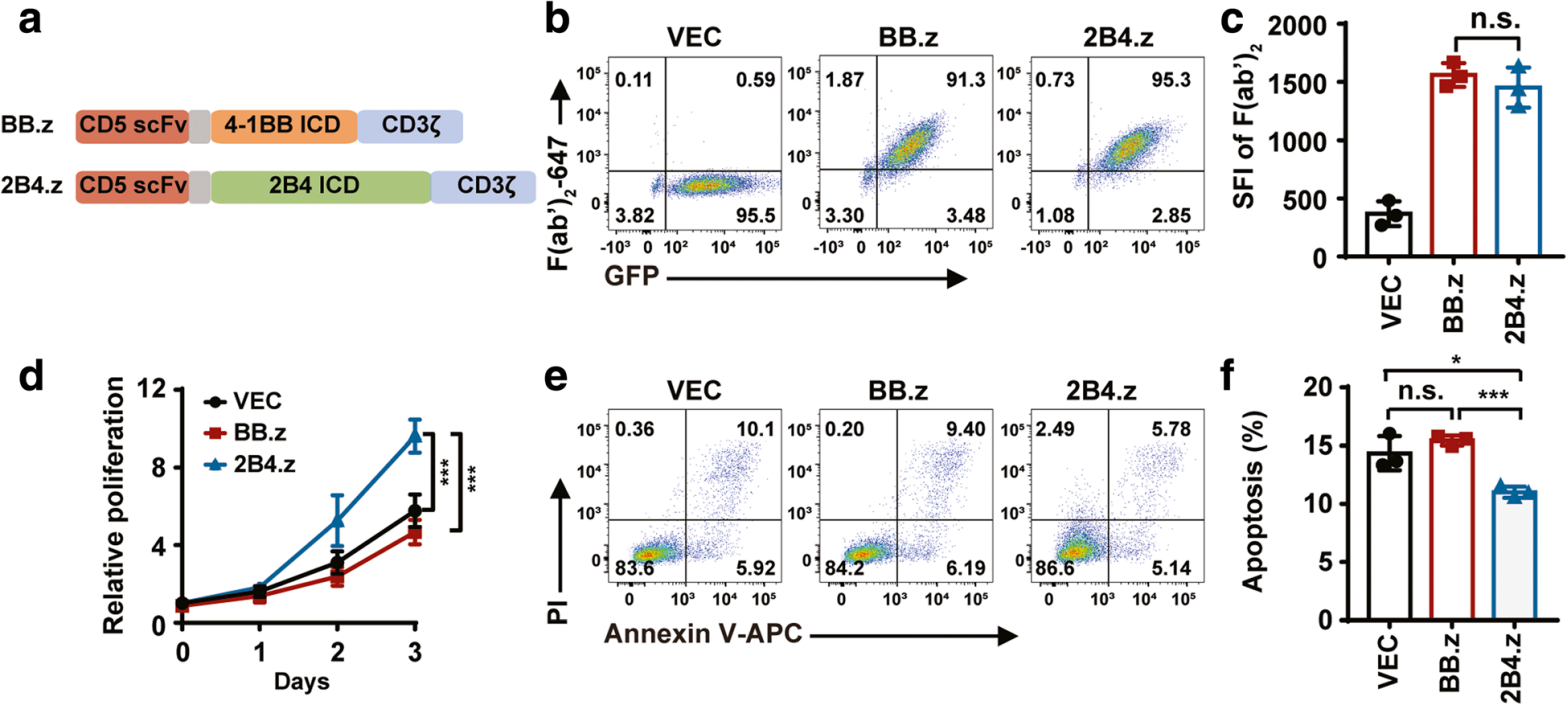
Construction of CD5 CAR and preparation of CAR-NK cells. **a** Schematic diagram of lentiviral CAR expression plasmids. **b** Representative flow cytometry analysis showing the expression of CARs on NK-92 cells. **c** SFI of F (ab’)_2_ on VEC-NK, BB.z-NK, and 2B4.z-NK cells (*n* = 3; n.s., no significance). **d** CCK8 assay of cell proliferation in VEC-NK, BB.z-NK, and 2B4.z-NK cells (*n* = 3; ***p < 0.001). **e** Representative flow cytometry analysis showing basal apoptosis of VEC-NK, BB.z-NK, and 2B4.z-NK cells. **f** Quantification and statistical analysis of the data in **e** (*n* = 3; *p < 0.05; ***p < 0.001; n.s., no significance). VEC: VEC-NK; BB.z: BB.z-NK; 2B4.z: 2B4.z-NK

To improve the cytotoxicity of CAR-NK cells against CD5^+^ hematologic malignant cells, two second-generation CARs with different costimulatory domains were generated, one with T-cell-associated costimulatory domain 4-1BB, referred to as BB.z, and the other with NK-cell-associated costimulatory domain 2B4, referred to as 2B4.z (Fig. 1a). NK-92 cells were infected with CAR structures lentivirus carrying CAR DNAs to generate CAR-NK cells. Then, CAR^+^GFP^+^ NK-92 cells were sorted and expended. To exclude the effects of infection efficiency and expression intensity of BB.z and 2B4.z on NK-92 cells, CAR-NKs with similar specific fluorescence intensity (SFI) were prepared (Fig. 1b, c). Surprisingly, after sorting GFP^+^CAR^+^ NK cells, rapid proliferation was found in 2B4.z-NK cells, which was confirmed by CCK-8 assay (Fig. 1d). Moreover, the 2B4 costimulatory domain attenuated the background level of apoptosis in 2B4.z-NK cells (Fig. 1e, f).

### 2B4.z-NK cells display superior anti-CD5^+^ hematologic malignant cell activity in vitro

**Fig. 2:**
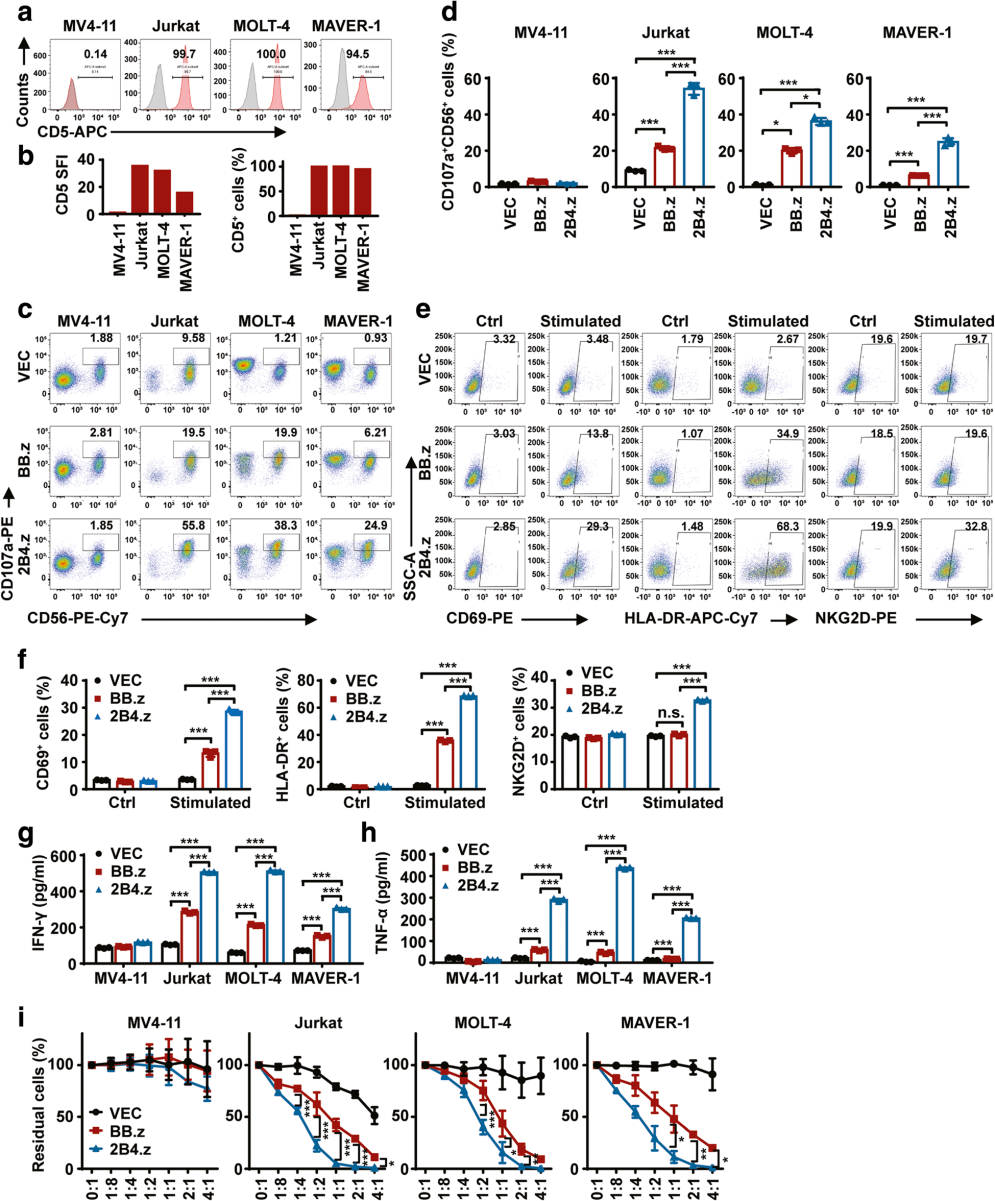
2B4.z-NK cells display superior anti-CD5^+^ hematologic malignant cell activity in vitro. **a** Representative flow cytometry analysis showing the expression of CD5 on MV4-11, Jurkat, MOLT-4, and MAVER-1 cells. **b** SFI (left panel) and proportion (right panel) of CD5 on MV4-11, Jurkat, MOLT-4, and MAVER-1 cells. **c** Representative flow cytometry analysis showing the proportion of CD107a^+^CD56^+^ cells after co-incubation with target cells as E:T = 1:3 for 5 h. **d** Quantification and statistical analysis of the data in **c** (*n* = 3; *p < 0.05; ***p < 0.001). **e** Representative flow cytometry analysis showing the expression of CD69^+^ (left panel), HLA-DR^+^ (middle panel), and NKG2D^+^ (right panel) cells in CD56^+^ cells after co-incubation with (stimulated) or without (Ctrl) MAVER-1 target cells for 6 h. **f** Quantification and statistical analysis of the data in **e** (*n* = 3; ***p < 0.001; n.s., no significance). **g** ELISA data showing the release of IFN-γ by NK cells after co-incubation with target cells for 12 h (*n* = 3; ***p < 0.001). **h** ELISA data showing the release of TNF-α by NK cells after co-incubation with target cells for 12 h (*n* = 3; ***p < 0.001). **i** Direct lysis of NK cells against target cells. Effector cells and target cells were co-incubated for 6 h at the indicated E:T ratio. Flow cytometry analysis of the percentage of CD5^+^CD56^−^ cells (*n* = 3; two-way ANOVA; *p < 0.05; **p < 0.01; ***p < 0.001). VEC: VEC-NK; BB.z: BB.z-NK; 2B4.z: 2B4.z-NK

To evaluate the cytotoxic effect of BB.z-NK, four target cells were used. MV4-11 cells were used as a negative control as they possess nearly no expression of CD5, while Jurkat, MOLT-4, and MAVER-1 cells were used as positive target cells with the proportion of CD5 positivity above 95% (Fig. 2a, b). The degranulation assay was used to detect the production of cytotoxic granules from NK cells and quantified by the expression level of CD107a [38]. After 5 h of coculture with MV4-11, no obvious degranulation was found in CAR-NKs or VEC-NK cells. When cocultured with CD5^+^ target cells, CD107a-positive cells were significantly increased in CAR-NK cells compared to VEC-NK. Encouragingly, 2B4.z-NK cells showed a remarkably higher percentage of CD107a-positive cells than BB.z-NK (Fig. 2c, d). In addition, CD69 [39], HLA-DR [40, 41], and NKG2D [42] are considered as activation markers of NK cells [43], and thus, the expression level of these three markers was assessed. The expression of these three activation markers on VEC-NK, BB.z-NK, and 2B4.z-NK cells remained at similar levels, without stimulation with CD5^+^ MAVER-1 target cells. After co-incubation with MAVER-1 cells, a moderate but significant increase in CD69 and HLA-DR expression on BB.z-NK cells was induced, while a dramatic increase in CD69, HLA-DR, and NKG2D expression on the surface of 2B4.z-NK cells was observed (Fig. 2e, f). Furthermore, IFN-γ [33, 44] and TNF-α [34], important cytokines for tumor surveillance and for inducing the activation of T cells and macrophages, are produced predominantly by NK cells and functionally linked to the cytotoxic activities of NK cells. Therefore, both IFN-γ and TNF-α cytokines were detected, revealing that CAR-NK cells could specifically secrete cytokines when cocultured with CD5^+^ cells and that the cytokines released by 2B4.z-NK cells were significantly higher than those released by BB.z-NK (Fig. 2g, h). Finally, the true lytic capability of CAR-NKs was evaluated by residual target cells, demonstrating that CAR-NKs could eliminate CD5^+^ malignant cells at a low E:T ratio (1:8) and that the cytotoxic capability of CAR-NK cells was significantly augmented by the 2B4 costimulatory domain (Fig. 2i). In brief, 2B4.z-NK cells exhibited higher cytotoxic activity towards CD5^+^ hematologic malignant cells than BB.z-NK cells in vitro.

### 2B4.z-NK cells exhibit cytotoxic activity against primary CD5^+^ hematologic malignant cells ex vivo

**Fig. 3:**
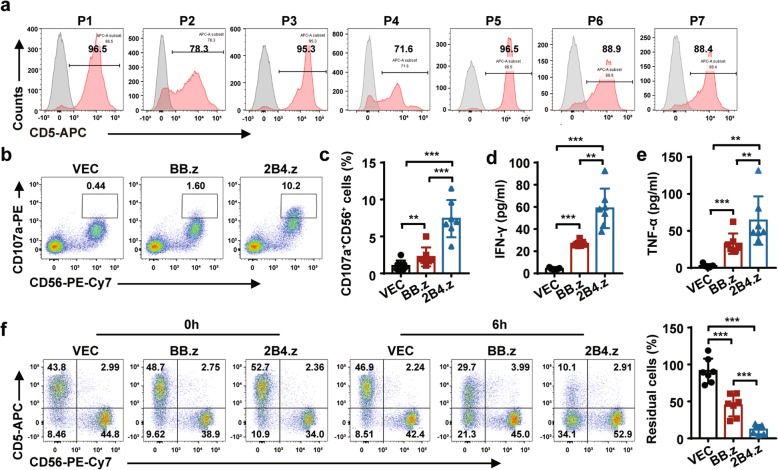
2B4.z-NK cells exhibit predominant cytotoxic activity against primary CD5^+^ hematologic malignant cells ex vivo. **a** Flow cytometry analysis showing the expression of CD5 on patients’ BMMNCs. **b** Representative flow cytometry analysis showing the proportion of CD107a^+^CD56^+^ cells after co-incubation with target cells as E:T = 1:3 for 5 h. **c** Quantification and statistical analysis of the data in **b** (*n* = 7; **p < 0.01; ***p < 0.001). **d** ELISA data showing the release of IFN-γ by NK cells after co-incubation with target cells for 12 h (*n* = 7; **p < 0.01; ***p < 0.001). **e** ELISA data showing the release of TNF-α by NK cells after co-incubation with target cells for 12 h (*n* = 7; **p < 0.01; ***p < 0.001). **f** Direct lysis of NK cells against target cells. Effector cells and target cells were co-incubated for 6 h at the indicated E:T ratio. Flow cytometry analysis of the percentage of CD5^+^CD56^−^ cells (left panel) and quantification and statistical analysis of residual cells (right panel) (*n* = 7; ***p < 0.001).VEC: VEC-NK; BB.z: BB.z-NK; 2B4.z: 2B4.z-NK

To further verify the cytotoxicity of 2B4.z-NK cells, seven patients’ primary CD5^+^ hematologic malignant cells were used in the study. CD5 expression on these primary hematologic malignant cells was analyzed by flow cytometry (Fig. 3a and Table 1). The median proportion of CD5-positive cells was about 88.9% (range 71.60–98.50%). Higher expression of CD107a (Fig. 3b, c), more IFN-γ (Fig. 3d) and TNF-α (Fig. 3e) release, and stronger specific cytotoxicity (Fig. 3f) were observed in 2B4.z-NK cells cocultured with CD5^+^ hematologic malignant cells. The results indicated that 2B4.z-NK cells were capable of recognizing CD5^+^ primary hematologic malignant cells and exhibited greater cytotoxicity efficacy than BB.z-NK cells.

**Table 1:** Patients information

Patient ID	Sex	Age	Disease	CD5(%)	SFI
P1	M	74	MCL	96.5	140.05
P2	F	65	CLL	78.3	207.71
P3	F	56	CLL	95.3	323.27
P4	M	73	CD5^+^ B-CLPDs unclassified	71.6	90.44
P5	F	67	CLL	96.5	109.07
P6	M	51	T-ALL	88.9	100.62
P7	F	58	T-ALL	88.4	83.36

*F* female, *M* male, *MCL* mantle cell lymphoma, *CLL* chronic lymphocytic leukemia, *CD5*^*+*^ *B-CLPDs unclassified* unclassified CD5^+^ B-cell chronic lymphoproliferative disorders, *T-ALL* T-cell acute lymphoblastic leukemia

### 2B4.z-NK cells have stronger anti-T-ALL activity in vivo

**Fig. 4:**
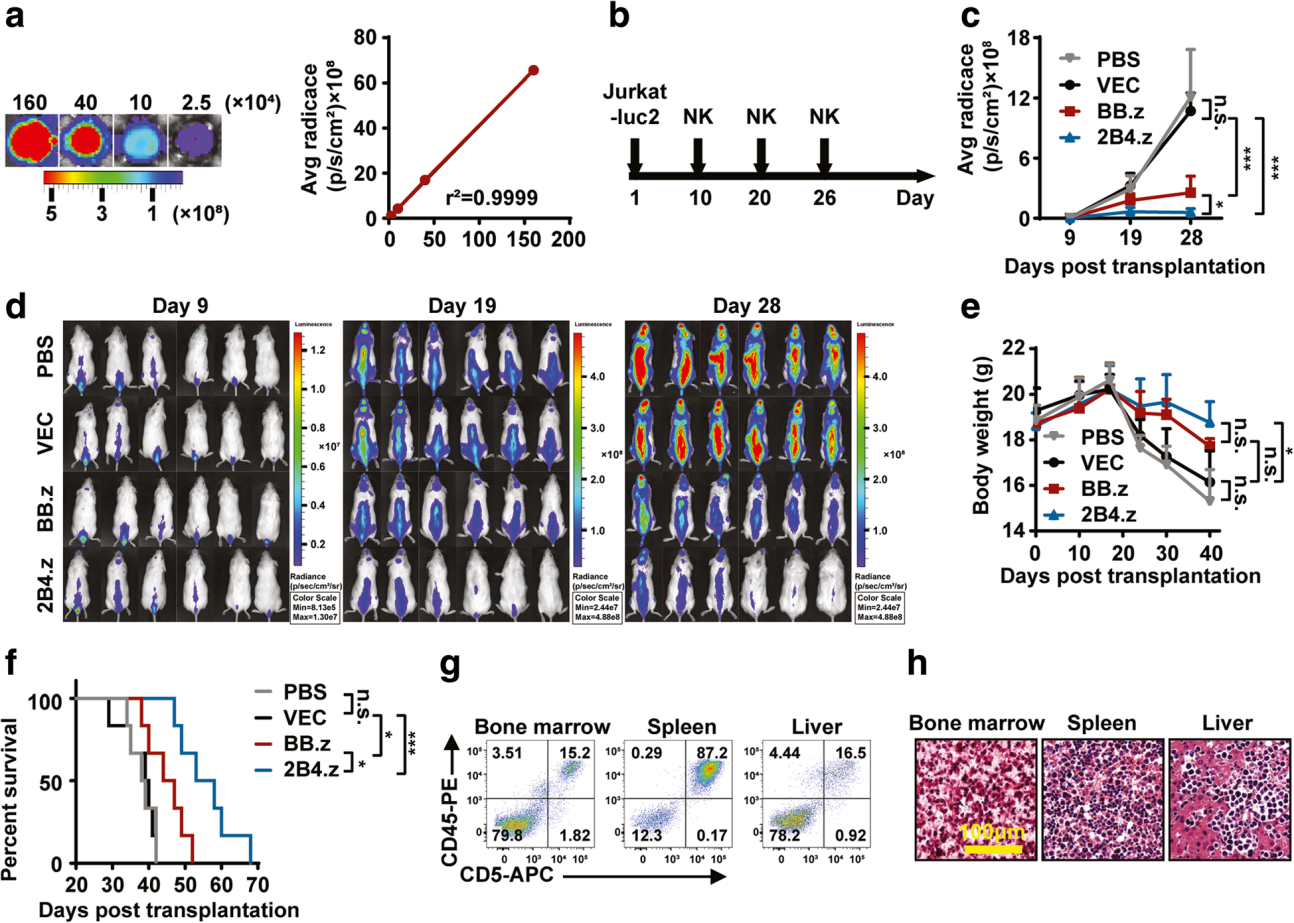
2B4.z-NK cells show stronger anti-T-ALL activity in vivo. **a** Jurkat-luc2 cells were seeded in 96-well plates at 1.6 × 10^6^, 4 × 10^5^, 1 × 10^5^, and 2.5 × 10^4^, 1.5 μl of 10 mg/ml D-Luciferin was added per well, and then bioluminescent images were obtained by using Caliper IVIS Lumina II. Left panel: representative bioluminescence images of Jurkat-luc2 cells; right panel: correlation analysis of bioluminescence signals and cell numbers (goodness of fit; *r*^2^ = 0.9935; *p* = 0.0033; *N*, number). **b** Schematic diagram of the treatment regimen. Mice were intravenously injected with 3 × 10^6^ Jurkat-luc2 cells. Nine days after transplantation, mice were divided into four treatment groups according to the average radiance of the bioluminescent imaging: group PBS, group VEC-NK, group BB.z-NK, and group 2B4.z-NK. Mice were respectively intravenously administered with PBS, 5 × 10^6^ cells of either VEC-NK, BB.z-NK, or 2B4.z-NK cells at day 10, day 20, and day 26. **c** Statistical analysis of the bioluminescence intensity of each treatment group measured at different days (*n* = 6; two-way ANOVA; *p < 0.05; ***p < 0.001; n.s., no significance). **d** Representative bioluminescence images of mice. **e** Body weight of each treatment group measured at different days (*n* = 6; two-way ANOVA; *p < 0.05; n.s., no significance). **f** Kaplan-Meier survival curves for mice (*n* = 6; log-rank test; *p < 0.05; ***p < 0.001;). **g** Representative flow cytometry analysis showing the proportion of CD45^+^CD5^+^ leukemia blasts in bone marrow, spleen, and liver of NSG mice. **h** Representative H&E staining of bone marrow, spleen, and liver of NSG mice.VEC: VEC-NK; BB.z: BB.z-NK; 2B4.z: 2B4.z-NK

To further the potential therapeutic application of 2B4.z-NK cells, their antitumor activities were investigated in a mouse model. A Jurkat cell line expressing firefly luciferase (Jurkat-luc2) was established, which showed a strong positive correlation (*r*^2^ = 0.9934) between firefly luciferase activity and cell numbers (Fig. 4a). Then, 3 × 10^6^ Jurkat-luc2 cells were systemically engrafted into immunocompromised NSG mice by intravenous inoculation. At days 10, 20, and 26 after transplantation, PBS or 5 × 10^6^ cells of either VEC-NK, BB.z-NK, or 2B4.z-NK cells were intravenously administered (Fig. 4b). Bioluminescence imaging was used to monitor Jurkat-luc2 cell growth (Fig. 4c). Compared with the mice treated with PBS or VEC-NK cells, Jurkat-luc2 cells were obviously suppressed in mice by CAR-NK cells treatment, especially in mice treated with 2B4.z-NK cells (Fig. 4d) as revealed by the decreased intensity of bioluminescence. The body weight of the mice indicated to some extent the state of disease progression. Twenty-four days after transplantation, the body weight of mice in the PBS and VEC-NK groups began to sharply decline until the death of the mice, while that of the BB.z-NK and 2B4.z-NK groups decreased steadily and slowly (Fig. 4e). Median survival times of the PBS, VEC-NK, BB.z-NK, and 2B4.z groups were 38.5 days, 39.5 days, 45.5 days, and 58.5 days (Fig. 4f), respectively, which was significantly extended in the CAR-NK groups. Among the CAR-NK groups, mice in the 2B4.z-NK group showed an even longer survival time compared to that of the BB.z-NK group (*p* < 0.05). There was no difference in the survival time between the PBS group and the VEC-NK group (*p* = 0.8342). All transplanted mice developed aggressive T-ALL with extensive infiltrations of Jurkat-luc2 cells in bone marrow, spleen, and liver, which was confirmed by flow cytometry (Fig. 4g) and pathological analysis (Fig. 4h).

### Both BB.z-NK and 2B4.z-NK have side effects on CD5^+^ normal T cells

**Fig. 5:**
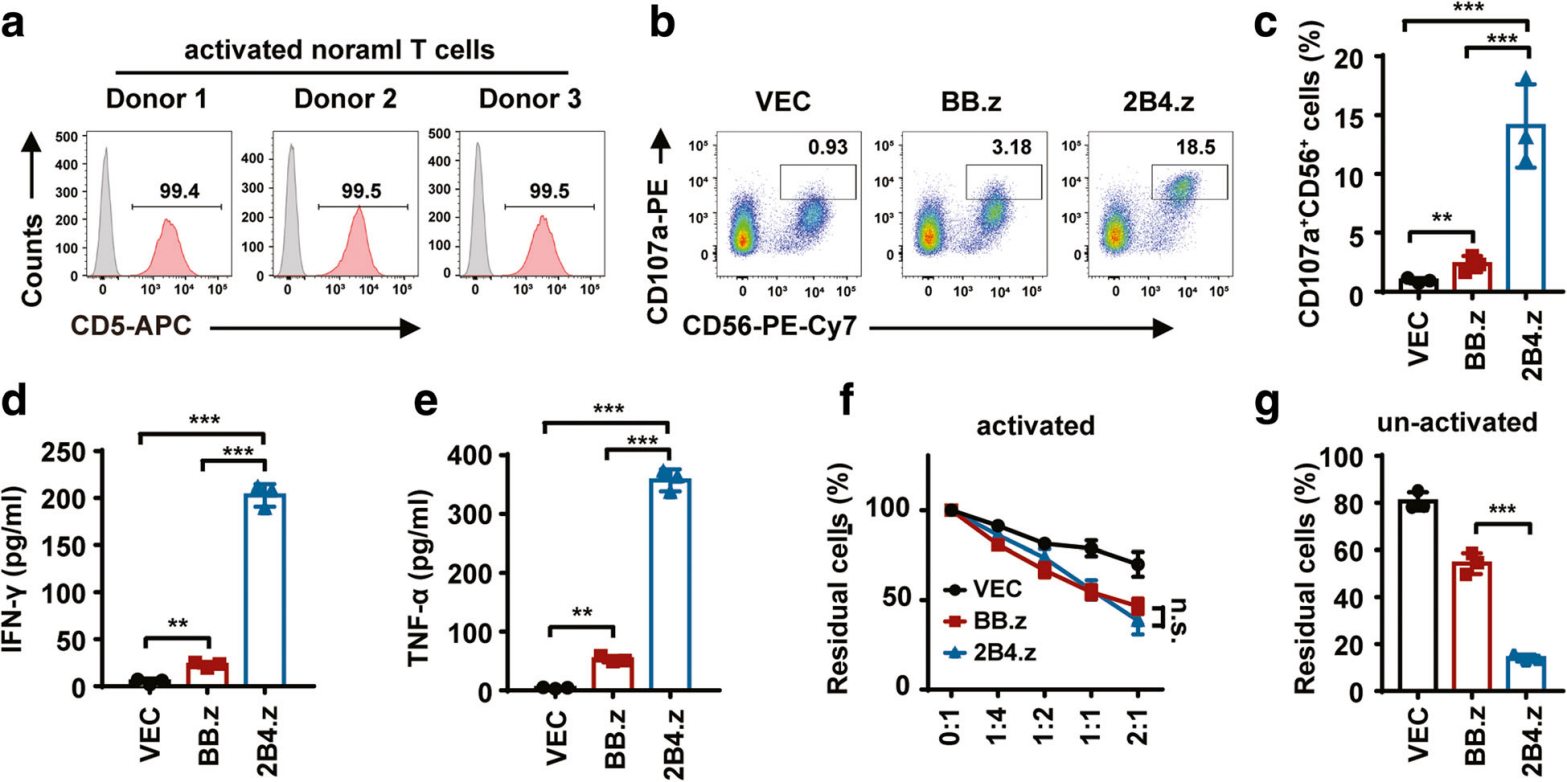
Both BB.z-NK and 2B4.z-NK present side effects on CD5^+^ normal T cells. **a** Flow cytometry analysis showing the proportion of CD5^+^ cells in donors’ normal T cells. **b** Representative flow cytometry analysis showing the proportion of CD107a^+^CD56^+^ cells after co-incubation with activated normal T cells as E:T = 1:3 for 5 h. **c** Quantification and statistical analysis of the data in **b** (*n* = 3; **p < 0.01; ***p < 0.001). **d** ELISA data showing the release of IFN-γ by NK cells after co-incubation with activated normal T cells for 12 h (*n* = 3; **p < 0.01; ***p < 0.001). **e** ELISA data showing the release of TNF-α by NK cells after co-incubation with activated normal T cells for 12 h (*n* = 3; **p < 0.01; ***p < 0.001). **f** Direct cytotoxicity of CAR-NK against activated normal T cells. Activated normal T cells and CAR-NK cells or VEC-NK cells were cocultured for 6 h at the indicated E:T ratio. Flow cytometry analysis of the proportion of CD5^+^CD56^−^ cells (*n* = 3; n.s., no significance). **g** Direct cytotoxicity of CAR-NK against un-activated normal T cells. Un-activated normal T cells and CAR-NK cells or VEC-NK cells were cocultured for 6 h at the E:T ratio of 1:1. Flow cytometry analysis of the proportion of CD5^+^CD56^−^ cells (*n* = 3; ***p < 0.001). VEC: VEC-NK; BB.z: BB.z-NK; 2B4.z: 2B4.z-NK

Finally, the side effects of CD5 CAR-NK on normal T cells were evaluated. In addition to expression on the surface of hematologic malignant cells, CD5 is also expressed on normal T cells [45]. Thus, three donors’ normal T cells were used for coculture with CD5 CAR-NK cells. First, the expression of CD5 on normal T cells was tested; the positive rate was above 99% (Fig. 5a). Then, T cells were co-incubated with VEC-NK, BB.z-NK, or 2B4.z-NK. The proportion of CD107a-positive cells was increased in both CAR-NK cells, while 2B4.z-NK cells showed more production of cytotoxic granules (Fig. 5b, c). CAR-NK cells, especially 2B4.z-NK cells, released a greater level of IFN-γ (Fig. 5d) and TNF-α (Fig. 5e) in the supernatant of the cocultured system. In addition, a true lysis assay of CAR-NK cells was performed, demonstrating that BB.z-NK and 2B4.z-NK had a similar cytotoxic ability against normal T cells (Fig. 5f and Additional file 1: Figure S1). Since the normal target T cells used for the above cytotoxic assay were activated by Human T-Activator CD3/CD28 Dynabeads and rhIL-2, these activated T cells proliferated rapidly, which might have obscured the true killing effect of 2B4.z-NK. Therefore, to exclude the effect of rapid proliferation of activated T cells on the CAR-NK killing effect, we further performed the true lysis assay using un-activated normal T cells as target cells and showed that the cytotoxicity of 2B4.z-NK cells against normal T cells was significantly higher than that of BB.z-NK (Fig. 5g).

## Discussion

T cell malignancies are aggressive hematological tumors with limited treatment strategies and dismal prognoses. To develop a CD19 CAR-T cell strategy for B cell malignancies, we investigated whether CAR engineered immune cells could exhibit a cytotoxic ability towards CD5^+^ T cell malignancies. Treatment of T cell malignancies using CD5 CAR-T cells remains limited due to the shared antigens between malignant T cells and normal T cells, causing the fratricide of CD5 CAR-T cells themselves. Recently, another important type of immune cell, the NK-92 cell, has been utilized as a CAR-modified immune cell. However, in preclinical models, CAR-T cells seem to be superior to CAR-NK-92 cells [7]. Therefore, we modified the CAR structure to improve the cytotoxic ability of CAR-NK cells.

In studies of CAR-T cells, the costimulatory domain has been considered an important factor that strongly affects the curative effect of CAR-T cells [9]. To date, nearly all engineered CAR-NK cells used the intracellular domain of T cell-associated costimulatory factors as the costimulatory structural module of CAR. Several studies constructed second-generation CARs with CD28 [43, 46] or 4-1BB (Clinical trial: NCT01974479 and NCT01974479) costimulatory domains, while others used third-generation CARs with both the CD28 and 4-1BB costimulatory domain [15, 47, 48]. At the beginning of our study, 4-1BB was used as a costimulatory domain of CAR, which has been proven effective in generating CAR-T cells to target CD20- [36], CD33- [35], and FLT-3-positive [49] malignant cells. The CD5 BB.z-NK cells in our preliminary study showed a good true killing ability against target cells (E:T = 1:1, 12 h), while the degranulation of BB.z-NK cells was not very obvious and the secretion of TNF-α was very low. This may due to the unsuitable function of 4-1BB in NK cells. Wilcox et al. reported that when they treated mice with 4-1BB ligand or anti-4-1BB agonistic antibody, proliferation was induced in NK cells and IFN-γ secretion increased alongside NK cell helper function, but the cytotoxic ability of NK cells was not augmented [50]. Several in vivo xenograft model studies have demonstrated that triggering the 4-1BB signaling of NK cells by treatment of mice with anti-4-1BB activating antibody [51] or interaction with 4-1BBL-positive γδT cells [52] would enhance NK-cell-mediated antibody-dependent cell-mediated cytotoxicity (ADCC) through the activation of the 4-1BB downstream signaling pathway. In contrast, the 4-1BB-4-1BBL interaction can attenuate the activity of NK cells in the human leukemia micro-environment. Several studies reported that 35% (23/65) of patients with acute myelocytic leukemia (AML) [53] and 32% (28/89) of patients with B cell chronic lymphocytic leukemia (B-CLL) [54] expressed a high level of 4-1BB ligand, and at the same time, almost all NK cells in these patients expressed 4-1BB. When 4-1BB ligand-positive AML cells interacted with 4-1BB on allogeneic NK cells, cytotoxicity and IFN-γ release were reduced, but this could be restored by 4-1BB blocking antibody [53]. When 4-1BB ligand-positive B-CLL cells interacted with 4-1BB on Rituximab-induced NK cells, ADCC was reduced [54]. These results are completely in opposition to those observed in T cells, where the interaction between 4-1BB and 4-1BB ligand would enhance the cytotoxic ability of human T cells against AML cells [55].

The different effects of 4-1BB between human NK and mouse NK cells, and between human NK and human T cells may be due to the different downstream signaling pathways induced by 4-1BB in these cells. The adaptor proteins TNF receptor-associated factor 1 (TRAF1) and TRAF2 will bind with the intracellular domain of 4-1BB (whether murine or human) after 4-1BB triggering [56], inducing the activation of the NF-κB, JNK, and p38 signaling pathway and leading to the activation of T or NK cells [57]. Because there are differences in several amino acids in the intracellular domain of human 4-1BB and murine 4-1BB, human 4-1BB can interact with another adaptor protein, TRAF3, whereas murine 4-1BB cannot [58]. When TRAF3 and TRAF2 form heterotrimers, they can inhibit NF-κB activation [59]. Therefore, the interaction between the 4-1BB costimulatory domain and the negative regulator-TRAF3 may result in the limited activation (low expression of CD107a and little releasing of TNF-α) of BB.z-NK, and thus, the downstream signaling pathway of NF-κB will be weakened.

In this study, we attempted to improve the cytotoxic ability of CD5 CAR-NK by changing the costimulatory domain of CAR. 2B4 is a member of the signaling lymphocytic activation molecule (SLAM)-related receptor family, which contain four immune-receptor tyrosine-based switch motifs (ITSMs) in their intracellular domain and perform important roles in regulating the reactivity of multiple immune cells [60]. The ligand of 2B4 is CD48, which is a glycoprotein-I (GPI)-anchored Ig-like protein that can be found in nearly all hematologic cells including NK cells [61]. Triggering 2B4 via interaction with CD48 can induce the phosphorylation of ITSMs, causing the recruitment of the adapter protein SLAM-associated protein (SAP) and EWS-Fli1-activated transcript 2 (EAT-2) [62]. SAP can recruit Src-family kinase Fyn [63], which then transduces downstream signals by phosphorylating phospholipase C-γ (PLC-γ) or Vav-1 [64], activating ERK, inducing the cytotoxicity of NK cells and producing the pro-inflammatory factors-IFN-γ and TNF-α. EAT-2 can link 2B4 to PLC-γ and ERK to mediate the activation of NK cells and accelerate the polarization and secretion of cytotoxic granules [65]. In one study of CAR-NK that used the 2B4 intracellular domain as the costimulatory domain, phosphorylation of PLC-γ, Vav-1, and ERK was promoted in NK cells [14]. It was revealed that the 2B4 intracellular domain may be more suitable as a costimulatory domain in CAR-NK cells than that of 4-1BB.

Therefore, in our study, we used the 2B4 intracellular domain as the costimulatory domain to replace the 4-1BB intracellular domain in the CAR structure and compared the cytotoxic ability of BB.z-NK and 2B4.z-NK towards CD5^+^ T-malignant cells. The results showed that 2B4.z-NK released more cytotoxic granules, expressed higher NK cell activation markers (CD69, NKG2D, and HLA-DR), secreted more of the inflammatory factors IFN-γ and TNF-α, and exhibited stronger true cytotoxicity than BB.z-NK after coculture with CD5^+^ cell lines and primary hematologic malignant cells in vitro. Moreover, 2B4.z-NK cells exhibited predominant cytotoxic activity on T-ALL bearing mice in vivo and significantly prolonged the survival of mice versus BB.z-NK.

In addition, CD5 is expressed in almost all normal T cells and some mature B cells [66]; thus, the side effects of CD5 CAR-NK were evaluated. The results showed that both BB.z-NK and 2B4.z-NK exhibited cell lysis properties, a side effect towards normal T cells (Fig. 5), while 2B4.z-NK revealed significantly higher cytotoxicity on normal T cells than BB.Z-NK cells, similar to their role in T cell malignancies. It is indicated that CD5 CARs targeting T cell malignancies will induce T cell aplasia similar to the B cell aplasia observed in patients treated with CD19 CAR-T cells. B cell aplasia is more resistant and can be relieved by immunoglobulin infusions [67]. Long-term T cell aplasia increases the probability of infection in patients [68]. Although 2B4.z-NK showed a stronger side effect towards normal T cells, the long-term T cell aplasia may be prevented by using short-lived CAR-NK cells or by bridging allogeneic hematopoietic stem cell transplantation after complete remission [7]. In our treatment strategy, the NK-92 cell line was used as the effector cells. For further clinical trial studies, 2B4.z-NK cells must be irradiated before transfusion into patients to prevent potential carcinogenicity. The process of irradiation will lead to the short-survival of 2B4.z-NK in patients, which can shorten the period of T cell aplasia in patients but may reduce treatment outcomes as well. To address this duality, multiple injections may be effective at prolonging the persistence of 2B4.z-NK cells in patients and augmenting the curative effect.

## Conclusions

CD5 CAR-NK cells, especially those constructed with the intracellular domain of NK-cell-associated activated receptor-2B4, exhibited specific cytotoxic properties against CD5^+^ malignant cells in vitro and remarkably prolonged the survival of T-ALL xenograft mice in vivo. 2B4.z-NK could be a potential immunotherapy strategy for T cell malignancy treatment.

## Additional file

Additional file 1: **Figure S1.** Both BB.z-NK and 2B4.z-NK present direct cytotoxicity on CD5^+^ normal T cells. (DOCX 728 kb)

## Abbreviations

ADCC: Antibody-dependent cell-mediated cytotoxicity
ALL: T cell acute lymphoblastic leukemia
AML: Acute myelocytic leukemia
B-CLL: B cell chronic lymphocytic leukemia
BMMNCs: Bone marrow mononuclear cells
CAR: Chimeric antigen receptor
CD5^+^ B-CLPDs unclassified: Unclassified CD5^+^ B cell chronic lymphoproliferative disorders
CLL: Chronic lymphocytic leukemia
CTCL: Cutaneous T cell lymphoma
EAT2: EWS-Fli1-activated transcript 2
ITSM: Immune-receptor tyrosine-based switch motif
MCL: Mantle cell lymphoma
NK: Natural killer
SAP: SLAM-associated protein
scFv: Single-chain variable fragment
SLAM: Signaling lymphocytic activation molecule
TCLs: T cell lymphomas
TRAF: TNF receptor-associated factor
