# Supplementary material for: 2B4 costimulatory domain enhancing cytotoxic ability of anti-CD5 chimeric antigen receptor engineered natural killer cells against T cell malignancies
Source: J Hematol Oncol. 2019 May 16;12:49. doi: 10.1186/s13045-019-0732-7 (PMC6524286; doi:10.1186/s13045-019-0732-7)
Supplement: Supplementary file 1 — Figure S1. Both BB.z-NK and 2B4.z-NK present direct cytotoxicity on CD5+ normal T cells. (DOCX 728 kb) [file 13045_2019_732_MOESM1_ESM.docx]

**Supplemental Information**

**2B4 co-stimulatory domain enhancing cytotoxic ability of anti-CD5 chimeric antigen receptor engineered natural killer cells against T-cell malignancies**

Yingxi Xu^1^ǂ, Qian Liu^1^ǂ, Mengjun Zhong^1^, Zhenzhen Wang^1^, Zhaoqi Chen^1^, Yu Zhang^1^, Haiyan Xing^1^, Zheng Tian^1^, Kejing Tang^1^, Xiaolong Liao^1^, Qing Rao^1^, Min Wang^1^*, Jianxiang Wang^1, 2^*

^1^State Key Laboratory of Experimental Hematology, Institute of Hematology and Blood Diseases Hospital, Chinese Academy of Medical Sciences & Peking Union Medical College, Tianjin 300020, China

^2^National Clinical Research Center for Blood Diseases, Institute of Hematology and Blood Diseases Hospital, Chinese Academy of Medical Sciences & Peking Union Medical College, Tianjin 300020, China

ǂ Equal contributors

*Correspondence to: Dr. Jianxiang Wang, E-mail: [wangjx@ihcams.ac.cn](mailto:wangjx@ihcams.ac.cn)

Dr. Min Wang, E-mail: [wangjxm@ihcams.ac.cn](mailto:wangjxm@ihcams.ac.cn)


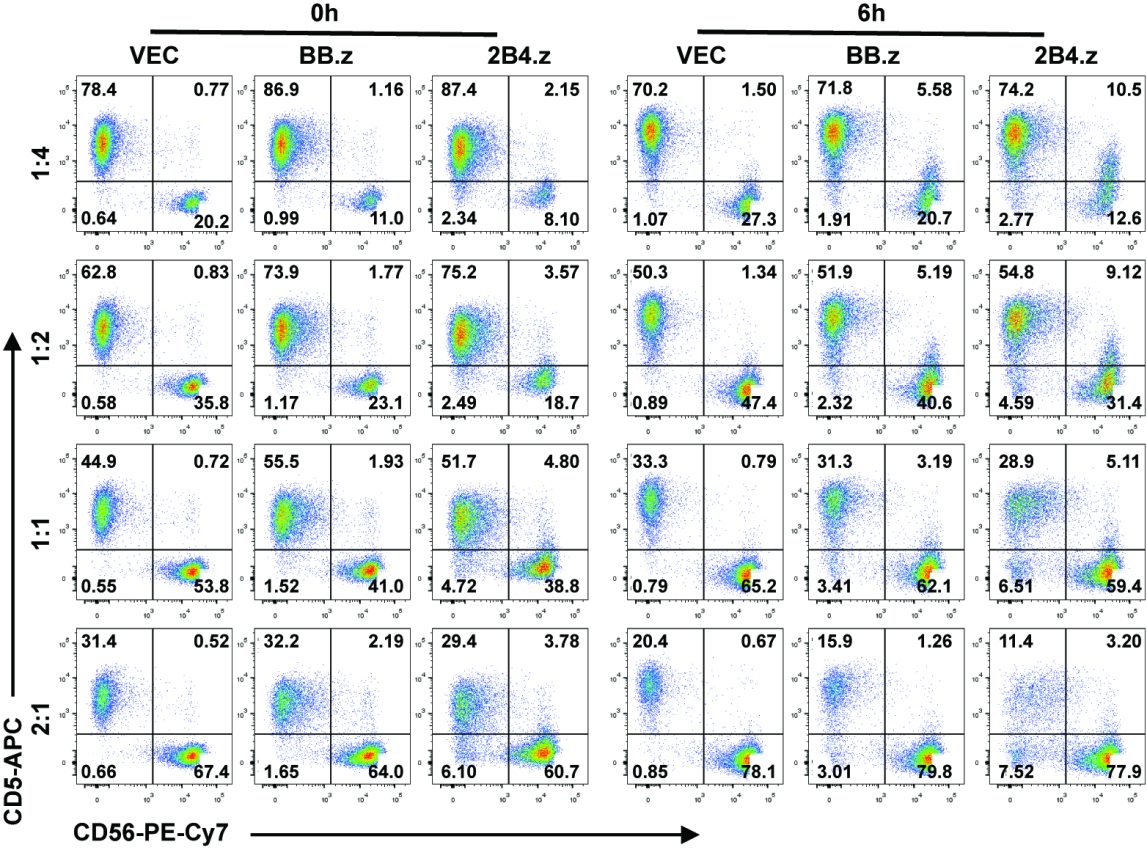


**Figure S1 Both BB.z-NK and 2B4.z-NK present direct cytotoxicity on CD5+ normal T cells.**

Normal T cells were isolated from peripheral blood of healthy donors, stimulated and amplified by using CD3/CD28 beads and rhIL-2 for 5 days, then followed by co-cultured with VEC-NK, BB.z-NK or 2B4.z-NK cells for 6h at the indicated E:T ratio. Flow cytometry analysis showing the percentage of the residual cells in the co-culture system.
